# Progress and Bottlenecks for Deep Learning in Computational Structure Biology: CASP Round XVI

**DOI:** 10.1002/prot.70076

**Published:** 2025-11-03

**Authors:** Andriy Kryshtafovych, Torsten Schwede, Maya Topf, Krzysztof Fidelis, John Moult

**Affiliations:** ^1^ Genome Center University of California California US; ^2^ Biozentrum University of Basel Basel Switzerland; ^3^ SIB Swiss Institute of Bioinformatics Germany; ^4^ Leibniz Institute of Virology and Centre for Structural Systems Biology Hamburg Germany; ^5^ Institute for Molecular Virology and Tumorvirology University Medical Center Hamburg‐Eppendorf Germany; ^6^ Institute for Bioscience and Biotechnology Research Rockville Maryland USA; ^7^ Department of Cell Biology and Molecular Genetics University of Maryland Maryland USA

**Keywords:** CASP, CASP16, community wide experiment, macromolecular ensembles, model accuracy, protein structure prediction, protein‐ligand complexes, RNA structure prediction

## Abstract

CASP16 is the most recent in a series of community experiments to rigorously assess the state of the art in areas of computational structural biology. The field has advanced enormously in recent years: in early CASPs, the assessments centered around whether the methods were at all useful. Now they mostly focus on how near we are to not needing experiments. In most areas, deep learning methods dominate, particularly AlphaFold variants and associated technology. In this round, there is no significant change in overall agreement between calculated monomer protein structures and their experimental counterparts, not because of method deficiencies but because, for most proteins, agreement is likely as high as can be obtained given experimental uncertainty. For protein complexes, huge gains in accuracy were made in the previous CASP, but there still appears to be room for further improvement. In contrast to these encouraging results, for RNA structures, the deep learning methods are notably unsuccessful at present and are not superior to traditional approaches. Both approaches still produce very poor results in the absence of structural homology. For macromolecular ensembles, the small CASP target set limits conclusions, but generally, in the absence of structural templates, results tend to be poor and detailed structures of alternative conformations are usually of relatively low accuracy. For organic ligand–protein structures and affinities (important for aspects of drug design), deep learning methods are substantially more successful than traditional ones on the relatively easy CASP target set, though the results often fall short of experimental accuracy. In the less glamorous but essential area of methods for estimating the accuracy, previous results found reliable accuracy estimates at the amino acid level. The present CASP results show that the best methods are also largely effective in selecting models of protein complexes with high interface accuracy. Will upcoming method improvements overcome the remaining barriers to reaching experimental accuracy in all categories? We will have to wait until the next CASP to find out, but there are two promising trends. One is the combination of traditional physics‐inspired methods and deep learning, and the other is the expected increase in training data, especially for ligand–protein complexes.

## 1 Introduction

CASP conducts community experiments to assess the state of the art for computational methods in structural biology. Experiments take place every 2 years. The CASP website (https://predictioncenter.org/casp16/) describes the process in detail. Briefly, participants are asked to calculate structures of biological macromolecules and complexes for which the experimental structures are not yet public. The results are assessed by independent assessors, and outcomes are published in a special issue of this journal, *Proteins*. This paper provides an overview of the results for CASP16, which took place in 2024. A total of 110 research groups from 22 countries used a total of 200 methods to address a range of computational structural biology areas. In all, over 128,000 submissions were received. Results were assessed for a total of 101 experimental targets provided by 56 groups from 15 countries. Forty‐eight of the target structures were obtained with X‐ray crystallography, 49 by cryo‐electron microscopy (cryo‐EM), and two by NMR. The other two targets are conformational ensembles and have only Residual Dipolar Coupling and SAXS data with no atomic co‐ordinates.

The 2020 CASP14 showed that the AlphaFold2 deep learning method largely solved the problem of computing the structure of protein monomers, often delivering results of comparable accuracy with experiment [1]. Since then, deep learning has been applied to most, if not all, areas of computational structural biology, and many different methodological variants have been proposed (e.g., as of September 2025, a PUBMED search for [“deep learning”] AND [“protein structure”] returned over 3500 hits). CASP has expanded its scope to incorporate some of the new areas, and inclusion of more is planned for future rounds. This report focuses on how successful deep learning was in CASP16 and discusses where future progress may emerge. Monomeric protein structures are still included in order to assess whether we are indeed as close to experiment as possible and to highlight situations where performance remains sub‐optimal. Protein complexes have been a separate category in CASP since 2014 [2], and it was again included in this round. Assessment is performed jointly with our sister organization CAPRI (Critical Assessment of PRotein Interactions [3]), providing two independent views of the results. Three new areas introduced as pilots in the 2022 CASP15, RNA structure and complexes, macromolecular multi‐conformational states, and protein–organic ligand interactions, are developed further here. Our analysis draws heavily on the work of the CASP16 assessors. They have done an enormous amount of work, and CASP could not function without them. References to the assessment papers are included in each section below. The large majority of methods used in this CASP, especially the relatively successful ones, incorporated one or more versions of AlphaFold: Multimer [4], AlphaFold2 [5], or AlphaFold3 [6]. The analyses show that in most categories the best overall group performance is slightly better than AlphaFold alone. Usually, that reflects the specifics of how AlphaFold technology was used (e.g., very extensive sampling [7, 8]; clustering generated structures [9] or combining an AlphaFold method with more traditional physics‐inspired approaches [10, 11]). The participant papers in this issue describe some of these methods, and the CASP16 abstracts (https://predictioncenter.org/casp16/doc/CASP16_Abstracts.pdf) provide some specifics for all approaches. Overall, the results show that AlphaFold3 [6] yields a modest improvement over AlphaFold2 [5].

## 2 Results

### 2.1 Protein Monomers

As noted above, the introduction of AlphaFold2 in the 2020 CASP14 resulted in computed monomer structures that in many cases appear to be as close to the corresponding experimental ones as possible, given experimental uncertainty [1]. Questions of most interest now are whether or not the structures are getting even closer to experiment and in what situations high accuracy models are not obtained. Here we summarize the main findings. Interested readers should see the official CASP16 protein monomer assessment paper [12].

**FIGURE 1:**
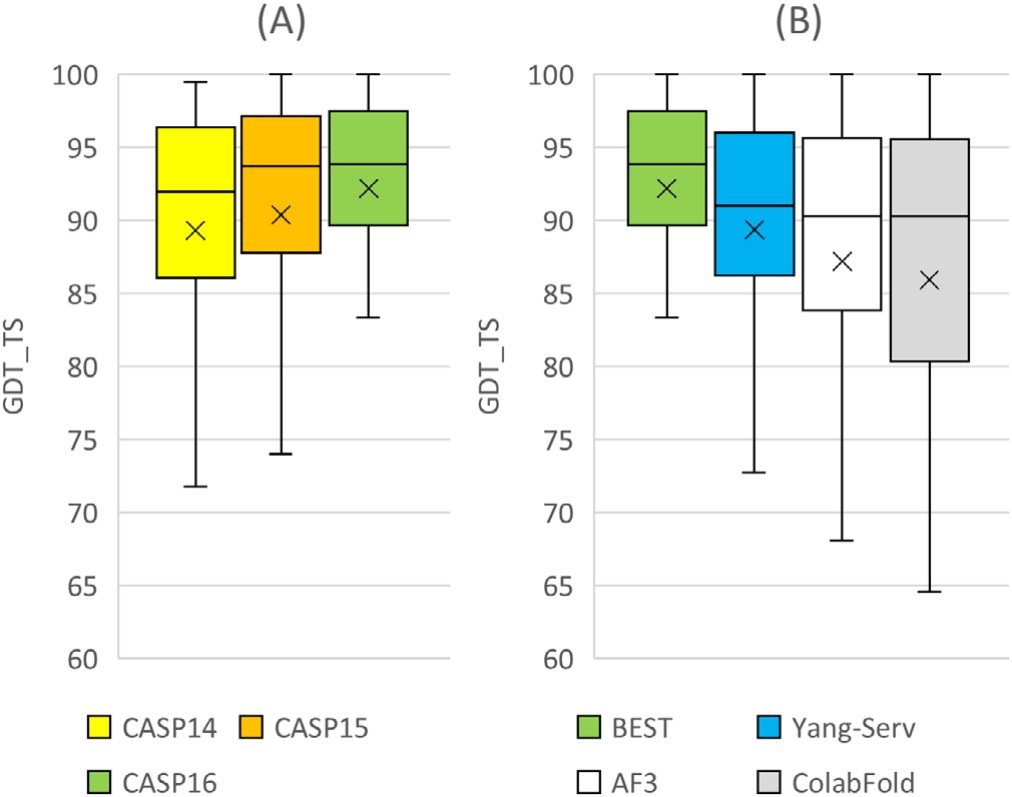
(A) Distribution of protein backbone agreement between calculated and experimental monomer protein evaluation units (EUs) for the three most recent CASP experiments, using the GDT_TS metric (max 100). In 2020 (CASP14), the introduction of AlphaFold2 resulted in a median value exceeding 90, and an average only slightly below 90. The subsequent 2022 and 2024 CASPs (15 and 16) show further minor improvement. For most targets additional improvement is likely limited by experiment accuracy. The fraction of poor scoring EUs continues to decrease. (B) Distribution of GDT_TS values for selected methods in 2024: The per‐EU distribution of highest scores from any participating group (green), the best‐ performing CASP16 method (blue), the AlphaFold3 server (white), and an AlphaFold2 method server (gray). No one method dominates; the most accurate method delivers better results than AlphaFold3, largely because of fewer poor scoring targets.

Figure 1A shows the distribution of GDT_TS [13] scores for main chain agreement between closest computed and experimental protein monomer evaluation units over the last three CASPs (2020, 2022, and 2024). In this CASP, 42 out of the 54 evaluation units are complete protein monomers, rather than smaller evaluation units (EUs) [14]. Where monomers are sub‐divided, the assessor found that all but two of the resulting EU interfaces are likely flexible, suggesting that the inter‐EU relationship may be environment‐dependent. There was a small improvement in accuracy between 2020 and 2022, but almost no further improvement in 2024, in spite of the introduction of AlphaFold3 [6], consistent with a saturation in possible agreement. Figure S1 shows the number of targets with closest agreement with experiment under different RMSD thresholds and supports that conclusion. There is a reduction in the number of poor scoring targets in 2024 though, reflected in the shorter lowest quantile segments. Figure 1B shows the agreement distributions in terms of GDT_TS for selected CASP16 groups. By this metric, the highest scoring individual group is Yang‐Server [15]. Agreement with experiment there is slightly lower than the best performance overall, indicating that no single group dominated performance. AlphaFold3 performs slightly less well than Yang‐Server. Yang‐Server placed major emphasis on multiple sequence alignment construction, together with use of AlphaFold methods. It is noteworthy that a server delivers the best performance—for much of the history of CASP, methods with human input performed better than servers. The models generated with the public AlphaFold3 server [16] show a similar median value but a lower average, reflecting more instances of relatively inaccurate models. ColabFold, a server running AlphaFold2 [17], has the same median score, but a lower average than AF3 as a consequence of a larger subset of low scoring targets—seven have more than a 10‐point GDT_TS drop between AF3 and AF2. Three of these are for viral proteins, suggesting this may be an area where AF3 is superior, perhaps because of less dependence on a robust multiple sequence alignment. Overall, group performance is improving by reducing the occurrence of poorer models, while not substantially increasing performance at the top end, again consistent with hitting the agreement limit.

What factors most influence the chance of getting a lower accuracy model? Two that have been suggested are not supported by the CASP data: in recent CASPs, the accuracy of membrane protein structures is indistinguishable from that of soluble proteins (Figure S2), and there is only a slight tendency for viral protein models to be less accurate than non‐viral ones (Figure S3). A caveat here is that the total number of membrane evaluation units over the last three CASPs is small (15). Also, although overall performance on viral proteins is comparable to that on others, some of the very lowest scores are for this class of target. Dependence of accuracy on alignment depth is minor (data not shown), but there are very few shallow MSA targets, so we may not see an edge effect. The biggest factor reducing accuracy seems to be protein size: for the 21 evaluation units with a best GDT_TS less than 90, only three are from targets smaller than 500 residues. One of those three is an experimental NMR structure, and for reasons that are not fully understood, over recent CASPs, NMR structure agreement is almost always lower.

As we have seen in recent CASPs, the computed structures are now of high enough accuracy that they can sometimes allow the experimentalists to correct minor errors in their initial structures. This time, comparison of experimental and computed model fits to experimental maps allowed the cryo‐EM assessor to offer advice on possible experimental structure improvements for three targets.

### 2.2 Protein Complexes

**FIGURE 2:**
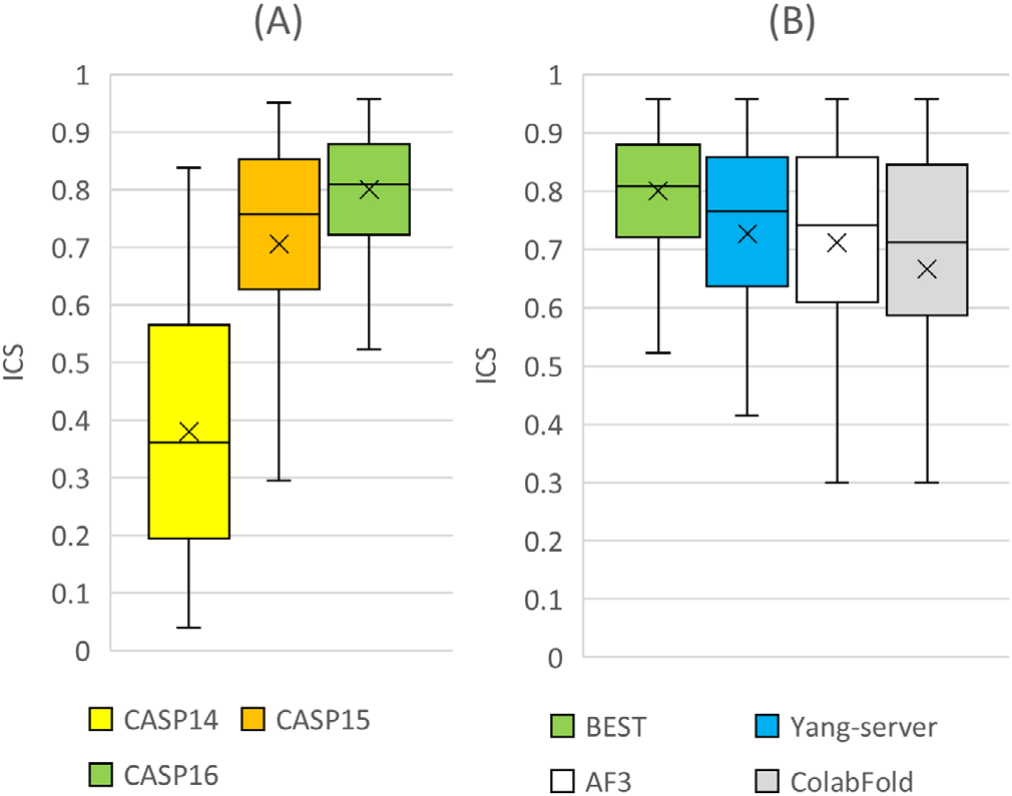
(A) Distribution of interface contact agreement between calculated and experimental interfaces in protein complexes across all targets for the three most recent CASP experiments, using an F1 metric (ICS). The major improvement from CASP14 to CASP15 reflects the successful use of deep learning methods. The further improvement in 2024 may be partly offset by an overall easier target set, although the reduction in the fraction of poor‐quality results is notable. (B) Distribution of interface ICS scores in the most recent CASP16 for the best scores from any group (green), the best individual group by this metric (Yang‐server, blue), the AlphaFold3 server (white), and an AlphaFold2 server (ColabFold, gray). No single group dominates the results. The slightly better performance of Yang‐server compared to AlphaFold3 likely reflects a more thorough treatment of multiple sequence alignments.

The CASP16 assessment of protein complexes [18] included 40 multimeric targets. Figure 2A shows performance in this category in terms of an F1 metric (ICS score) [19] for inter‐subunit contact agreement between calculated and experimental structures. There is a huge increase from CASP14 to CASP15, directly attributable to the introduction of AlphaFold deep learning methods. There is an apparent smaller increase from CASP15 to CASP16, although the assessor finds that some of the gain may reflect a slightly easier target set [18]. The median and average contact accuracy scores are both around 0.8. We have no data on the upper limit imposed by finite experimental accuracy, but it is likely higher. Thus, it appears that although deep learning has resulted in a very large improvement, experimental accuracy has not yet been reached.

Figure 2B shows the distribution of highest per‐target interface contact scores obtained by participating groups, the best individual method by this metric, Yang‐server [15], the AF3 server [16], and the AF2 server (ColabFold [17]). As with the protein monomer results, the overall best scores for each target are higher than those of the single best group, indicating that multiple groups contributed highest scores, depending on the target. Also, as with monomers, the best single group performed slightly better than AF3, and the AF3 distribution is a little improved over AF2.

As in the previous CASP, one class of target in particular was challenging; that is, antibody‐ and nanobody‐antigen complexes, likely because of the absence of cross‐interface evolutionary information.

**FIGURE 3:**
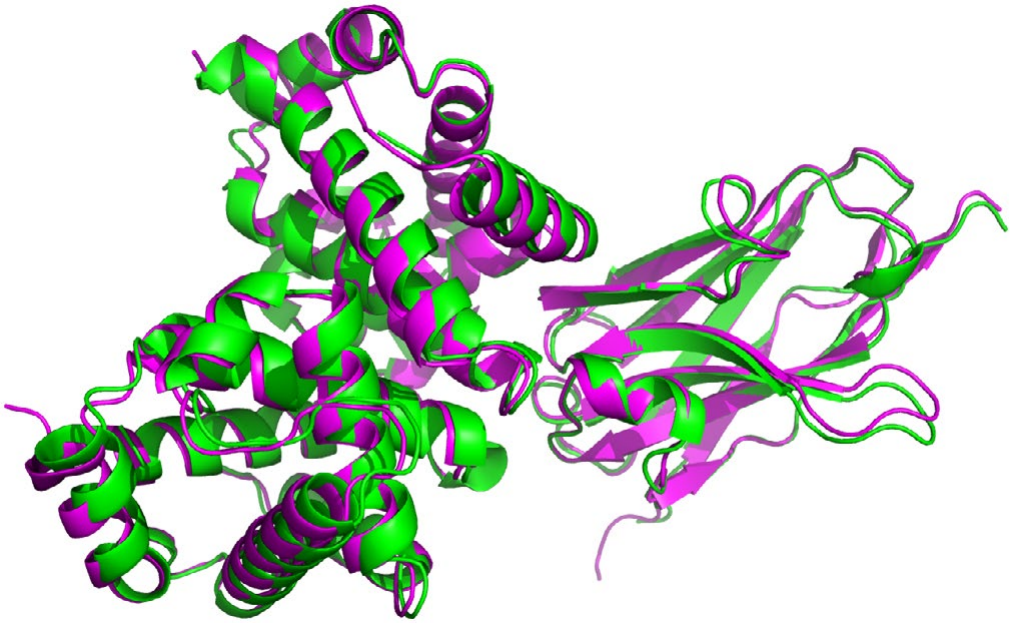
Success with an immune complex. Superposition of the experimental (green) and computed (purple) structures of a Llama‐derived nanobody and human hemoglobin (Target H1204). Immune complexes are generally still problematic for current methods, but new approaches show promise. This structure was obtained using a combination of large‐scale sampling, a physics‐based method, and various versions of AlphaFold.

In the previous CASP, we saw hints that some methods could sometimes overcome this difficulty. In particular, the Wallner group obtained the best performance [20] using an extensive sampling protocol, with multiple seeds, more recycles, variations in the multiple sequence alignment, and network layer dropout. This time, the kozakovvajda group had an impressive (though still only partially successful) performance, achieved by a combination of their traditional physics‐based docking method [10] with extensive sampling and refinement using various AlphaFold versions. Figure 3 shows an example of a successfully modeled complex. Evidently, the diversity of initial poses and samples allowed AlphaFold methods to be more successful overall. However, scores are poor for three of the eight targets in this category, and it is not clear how much of the success is attributable to the traditional approach. Still, this is an encouraging result.

In previous CASPs, stoichiometry information was always provided for protein complex targets, and the results outlined above were obtained under those conditions. In a normal research setting, that information may not be available. In CASP16, stoichiometry was initially not provided. For ~70% of targets, many groups were able to identify and use the correct stoichiometry (usually from related complexes). For more difficult cases, some groups successfully tested multiple stoichiometries.

As noted above, in the previous CASP, a carefully crafted sampling protocol using AF2 [7] proved advantageous. In this CASP, the MassiveFold group [21] provided sets of ~8040 models for many targets, generated using an expanded version of the Wallner sampling protocol. The model sets were provided to participants as an additional step for each target. In general, that did not lead to better agreement with experiment, but the data did allow some of the less successful groups to improve their performance. Some MassiveFold analysis is included in the section on model accuracy estimation, below.

### 2.3 RNA and Related Structures

**FIGURE 4:**
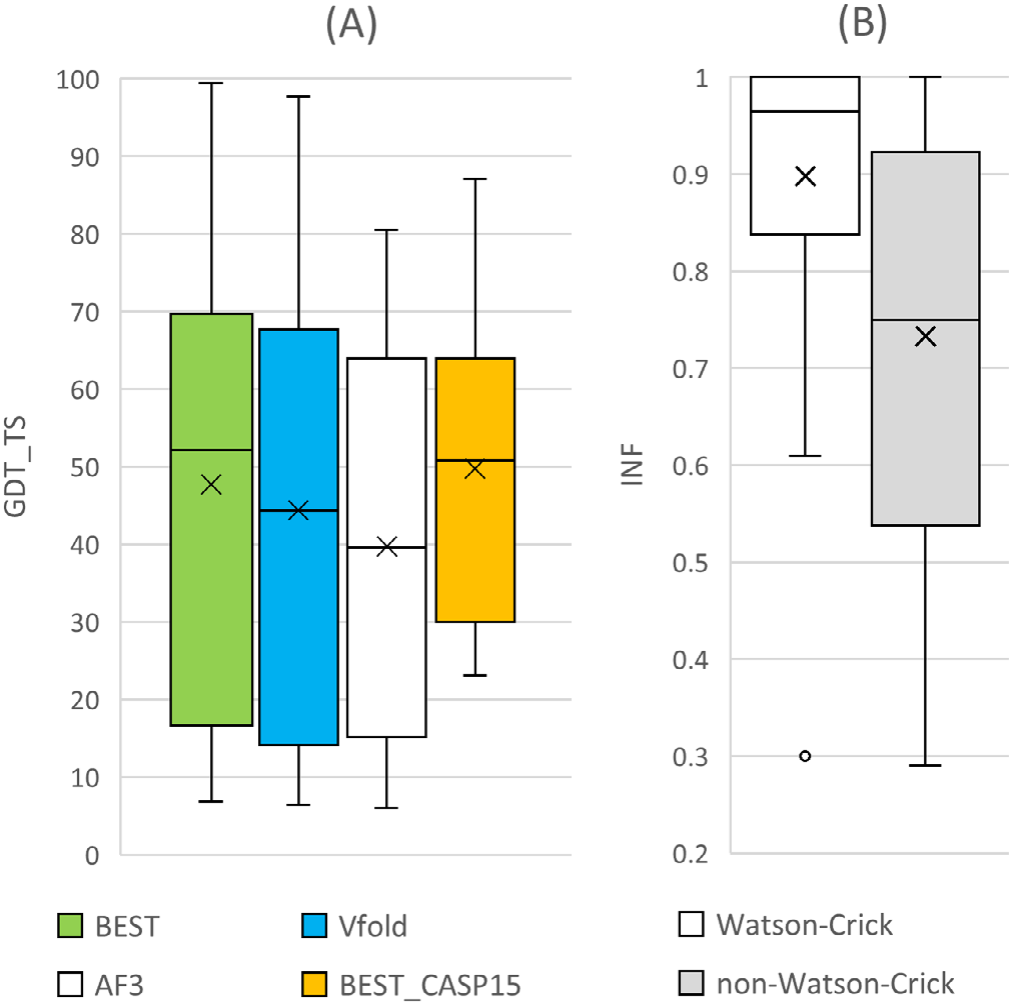
(A) Distribution of highest backbone agreement between calculated and experimental structures across all RNA monomers for selected groups in the most recent two CASP experiments, using the GDT_TS metric (max 100). The CASP16 distributions of highest scores for each target (green), the best‐ performing method by this criterion (Vfold, blue), the AlphaFold3 server (white) and the highest scores per target in the preceding CASP (yellow) are shown. The average and median scorers are poor and are dragged down by very poor performance where there is no structural template available (data not shown). The best single group performance is lower than the overall best, indicating no single group dominates. AF3 server performance is lower than that of the best group (see text), There is no apparent improvement over the previous CASP. (B) Distribution of base‐pairing agreement with experiment over all RNA monomer targets, using the INF metric [24]. Success is high for standard Watson‐Crick base pairs, and much poorer for non‐canonical pairing (see next figure).

**FIGURE 5:**
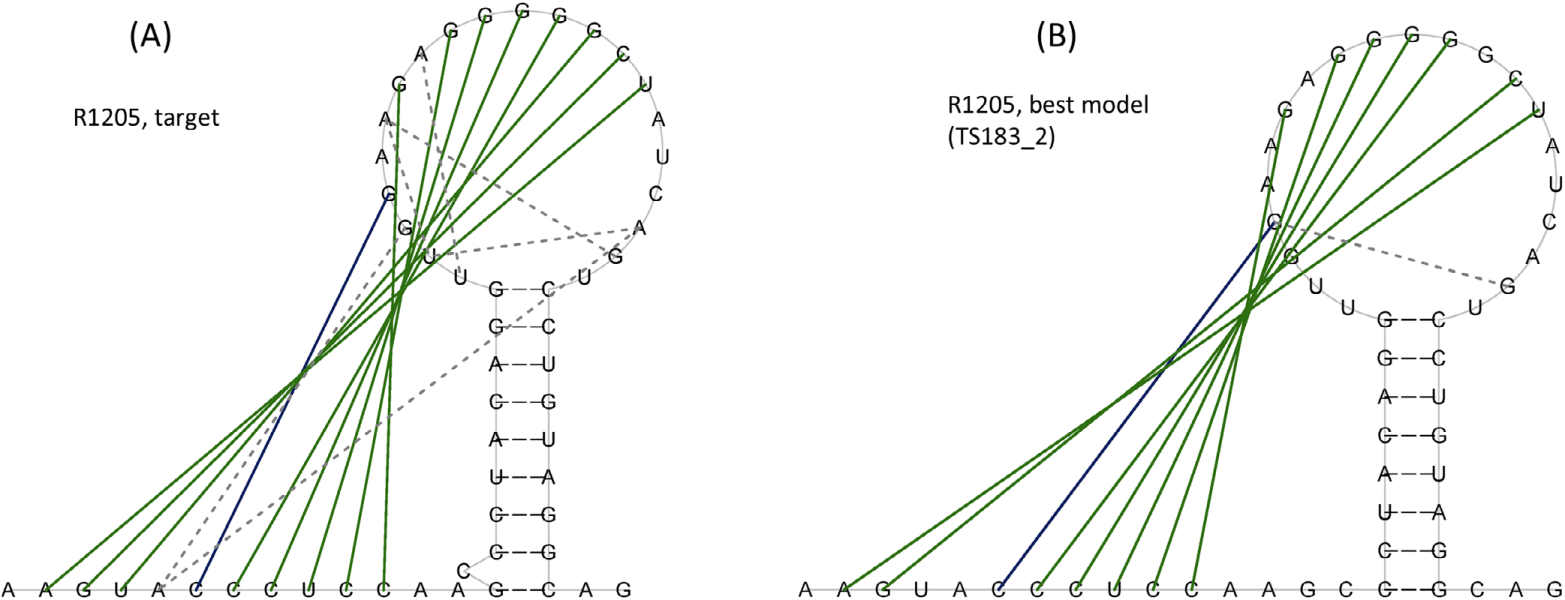
Comparison of base pairing between experiment (A, left) and the closest computed structure (B, right) for RNA target R1205. Long‐dashed and solid lines indicate canonical Watson‐Crick base pairs (colors of solid lines represent different nested pseudo‐knot sets); short‐dashed lines represent non‐canonical base pairs. Typical of the overall results, Watson‐Crick pairing is captured well (two discrepancies), but none of the six experimentally observed non‐canonical pairings is present in the computed structure, and an additional non‐canonical pair is introduced. (Diagrams are generated with RNApdbee 3.0 [27] using FR3D base‐ pairing algorithm [24] and RNApuzzler visualizer [28]).

The RNA structure category was introduced in the 2022 CASP15, with the hope that the deep learning methods that are so successful for proteins could be adapted to this domain. There was already a large body of work in developing physics‐inspired approaches, and RNA‐Puzzles [22] has run a series of structure challenges in this area. In CASP15, although the number of targets was small, the results suggested that deep learning was not yet effective [23]. With more targets (42) in the recent CASP, that conclusion still holds: in the absence of a related structure to provide a template, almost all submissions are of poor accuracy (Figure 4A) and the best deep learning performances are still not better than those of traditional methods. Analogous to the procedure for protein complexes, there were two independent assessments, one by Eric Westhof and his colleagues [25] using the RNA puzzles approach and the other by Rhiju Das, Rachael Kretsch, and colleagues [26]. The assessors found that whereas most of the standard Watson‐Crick base pairs were reproduced, often resulting in the double helical portions of structures being accurately modeled, other sorts of canonical base pairing were less accurate, and non‐canonical pairs were very poorly reproduced (Figures 4B and 5).

One promising development is that the best group performance (Vfold) used a combination of a traditional method with deep learning [11]. Whether this hybrid approach can be further developed to provide substantially more accurate structures remains to be seen.

Sixteen of the RNA targets are complexes, with RNA, protein, and in a few cases DNA binding partners. As expected, given the poor quality of the RNA monomer components, accuracy for these was also poor [18, 26].

Why is deep learning less effective for RNA? These are very different macromolecules from proteins with different inter‐residue interactions, possibly making the problem inherently more difficult. But the more likely primary reason is the much lower number of RNA structures in the PDB compared with proteins, and hence lower training power.

### 2.4 Protein–Organic Ligand Complexes

Computational methods for modeling the binding pose and estimating the binding affinity of small organic molecules to proteins are very relevant to small molecule drug design, particularly for finding classes of small molecules that bind and for the refinement of those into lead compounds. As a result, there is a long history of methods development in this area. D3R conducted community assessments for some years [29], but is no longer active. In the 2024 CASP, for the first time, we obtained four challenge data sets from pharma companies: three from Hoffman La Roche: 17 chymase complexes, two cathepsin G complexes, and 189 autotaxin complexes; and one from Idorsia Pharmaceuticals: 20 Mpro complexes. These are all compound refinement data sets—the proteins are well‐known drug targets and have some ligand complexes available in the PDB. Some of the structural data have accompanying affinity data. The assessment is reported in [30].

**FIGURE 6:**
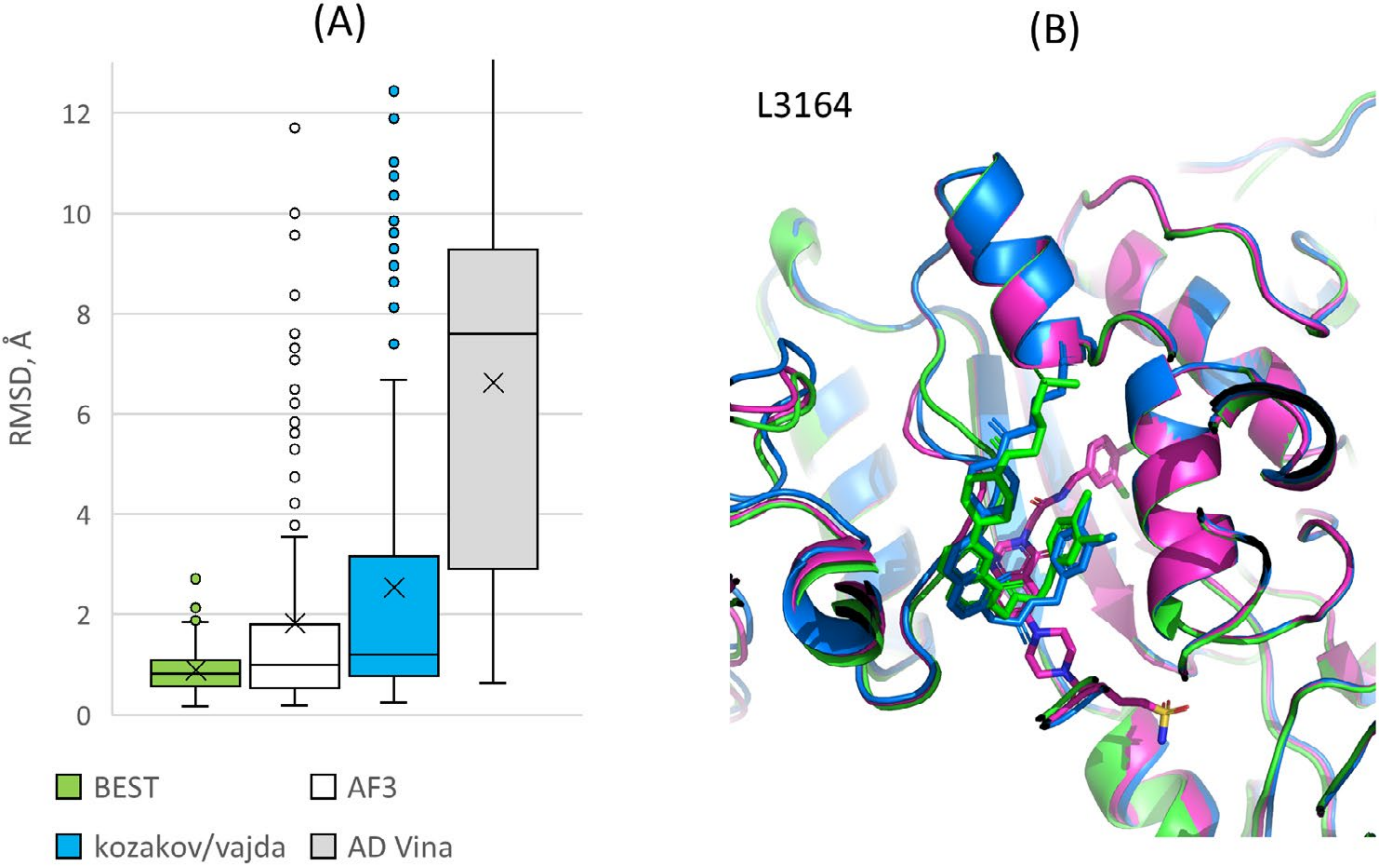
(A) Distribution of root mean square all‐atom difference between computed and experimental ligand poses for the best results obtained for each target (green), AlphaFold3 (white), those of the best‐ performing group by this metric (kozakovvadja, blue), and a non‐deep‐learning method (Autodock Vina, gray). AF3 has the strongest performance, although there is a substantial number of failures. The best of any group distribution indicates that one or more other groups did produce more accurate models for those outliers. (B) Example of a complex of autotaxin with a ligand (L3164) where AF3 produced a poor pose (magenta), but at least one other group was more successful (blue, DIMAIO). The experimental structure is in green.

Figure 6A shows the distribution of atomic root mean square deviation for the best scores received on each target, for those from AlphaFold3 (run as a control), the best‐ performing participating group by this metric (kozakovvajda [10]), and a second control with a popular traditional method, AutoDock Vina [31]. Here, AF3 comfortably outperforms all participating groups. On the other hand, the traditional method has relatively weak results, primarily because of poor performance on one of the four datasets. An interesting feature of the results is that for almost all targets at least one group submitted an accurate model, whereas most individual methods have a substantial number of poor results (the outlier points at the top of the distributions). Figure 6B shows an example that suggests that if reliable accuracy estimates are available, running multiple methods could yield better results in practice.

How good are these results in application terms? Practitioners in the computational field have adopted 2.5 Å RMSD as the threshold for “success” [30]. However, it is unlikely that chemists selecting the next compound to synthesize would be happy with that, and a 1 Å threshold seems more realistic. The AF3 results have a median RMSD just over 1 Å, suggesting roughly half of the AF3 results are adequate, and the other half are not. Also, there is a large number of outliers, pulling up the average to nearly 2 Å. As noted above, outlier cases do not seem to be intrinsically intractable. Overall, the performance of AF3 and other high‐ scoring groups is encouraging. At least for these datasets, the results are a large advance over the traditional control method. An important caveat is that there are structural fragment templates for many of these compounds in the PDB, as well as known protein structures and complexes, likely aiding performance. A recent benchmarking study for this type of method found that performance degrades dramatically for novel complexes [32]. The CASP16 test set includes just one novel target where novel classes of compounds are sought, an outcome of a CACHE community experiment [33]. It happened that for this case, one group predicted an accurate pose.

In contrast to the structure results, prediction of affinities was very unsuccessful, with a Kendall's *τ* of just 0.42 [30]. A second round of affinity prediction, where participants were given the experimental structures of the complexes, did not improve performance.

### 2.5 Estimated Model Accuracy

**FIGURE 7:**
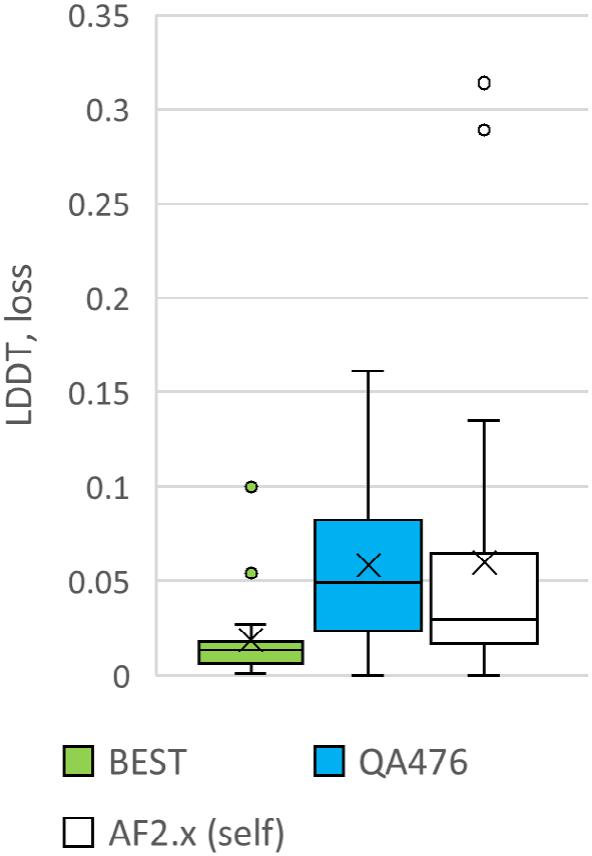
Loss of accuracy in choosing the predicted most accurate monomer structure out of the ~8040 models per target provided by MassiveFold for 24 targets where the MassiveFold range is greater than 0.2 overall LDDT units. For most targets, at least one participating group selected a model close to optimum (Best, green). Best‐ performing groups, such as the one shown (QA476 VifChartreuse, blue), have low median losses of about 0.05, but by this measure are substantially worse than the self‐estimates provided with the AlphaFold2 models (white).

In many areas of science, it is usual to provide accuracy estimates for any reported data, and that should of course be the standard in computational structural biology as well. CASP has a well‐established category for this. Accuracy estimates for overall structures and single residues have increased in reliability, particularly those provided with the submitted models [34]. As noted earlier, in this CASP, the MassiveFold group provided sets of several thousand models per target, providing a new opportunity to test accuracy estimation methods. Groups were asked to identify the five most accurate structures (as measured by closeness to experiment) for both monomers and complexes. Figure 7 shows the monomer results for non‐trivial targets (those where model accuracy spanned a range of at least 0.2 LDDT points; see Figure S4). Many of the more successful methods used the pLDDT from the MassiveFold models, or slight modifications of those. But some groups used independent methods: for example QA476 VifChartreuse, shown in the figure, used a version of the Voronoi tessellation [35] approach. Judged by loss of accuracy in choosing the predicted most accurate structure versus the actually most accurate of the set, such independent methods were quite successful, with the best ones having a median loss of ~0.05 LDDT. However, the self‐estimates provided with AlphaFold2‐generated structures have the lowest median loss (~0.02). This result reinforces conclusions from previous CASPs that it is difficult for third‐party methods to outperform accuracy estimates integral to model generation.

**FIGURE 8:**
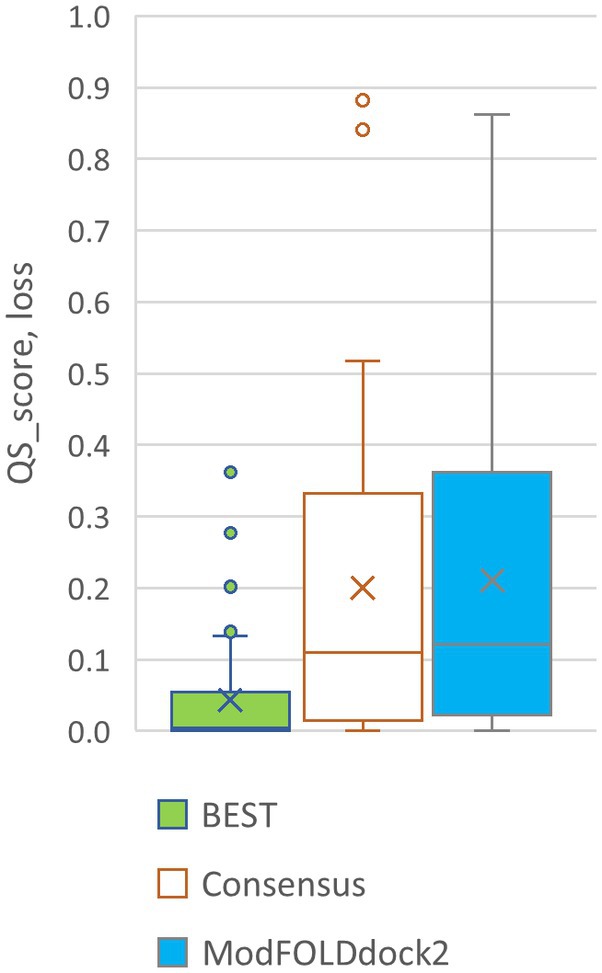
Reliability of methods for estimating the accuracy of protein–protein interfaces, in terms of the loss of agreement with experiment in choosing the computed structure with the highest predicted interface accuracy instead of the one with the actual highest accuracy, using the QS‐score metric. For most targets, at least one group assigned the highest accuracy to a model very close to optimal, so that the distribution for the best of any group (green) has a median close to zero, with a few large failures. A consensus method that averages the QS‐score differences between a reference complex and all other submitted models performs much worse, but still the median loss is only ~0.1 on the QS‐score scale. The best participating method's (ModFolddock2, blue) has a slightly worse performance than the consensus. (QS‐score is the normalized fraction of shared interface contact residues with a Cβ–Cβ distance < 12 Å between a computed and experimental structure [36]).

Accuracy estimates for complexes are less well developed, and AlphaFold methods do not provide interface accuracy estimates. This CASP again included an assessment of overall accuracy estimates for protein–protein interfaces. The estimates were judged in terms of the difference between the interface scores of models selected as the most accurate; that is, those with the highest predicted QSCORE [36] and the one that is in fact closest to experiment according to the QS‐score. Figure 8 shows that, while almost always at least one participating group picked a close to optimum model, no single group performed at that level of accuracy, and the best performance is slightly worse than a simple consensus method [34]. In practice, a consensus method that requires 100s of models is not useful. The best individual method (ModFOLDdock2 [37]) uses a neural network to generate accuracy estimates, with input from 12 individual single methods, some of which use a small‐scale consensus approach. There is more work to do in this area. Full details of the accuracy estimation assessment are in [38].

### 2.6 Conformational Ensembles

Anfinsen's original statement of the protein folding problem [39] assumed that one protein sequence adopts a single‐folded, functional conformation. We now know that many proteins have multiple functional states, and so do other macromolecules, such as folded RNA molecules. Further, many portions of protein molecules and probably the vast majority of RNA molecules do not have any highly populated conformations, at least in the absence of binding partners. Rather, in the jargon of the field, they are intrinsically disordered. In some cases, intrinsically disordered regions are critical to function, for example in mediating liquid–liquid phase separation [40]. In others, they may simply act as spacers between functional units, and the exact conformational ensemble they adopt is only of academic interest with no functional implications.

Given the success of deep learning methods for single protein conformations, a natural next question is to what extent those and other approaches can model multi‐conformational states. This is a matter of controversy [41], partly centered around whether success is seen only in cases where all relevant conformations are present in the training data. The 2022 CASP pilot in this category produced mixed results on a small number of targets, with one case of a previously unseen domain swap induced by a single mutation being successfully predicted by several groups [42]. In CASP16, we attempted to obtain a larger and more representative target set, but were only partially successful. Assessment [43] in this category was complicated by several factors. First, for almost no targets were high accuracy (in the sense of agreement with experiment) computed structures obtained. That may be the main conclusion from the study: for the CASP16 targets, with one exception, no methods are competitive with experiment. Second, the existence of structurally similar conformations in the PDB makes it difficult to judge whether template information is utilized in some way to deliver the best‐scoring models. Third, some structures may have a broader range of conformations than happen to have been captured experimentally. That is, a submitted structure may in fact be part of the conformational ensemble that has not yet been observed experimentally (see for example, Figure S5). Fourth, some targets include DNA components, and the results suggest that, like RNA [26], the prediction of these is still a largely unsolved problem.

Of the 10 targets considered to have multiple distinct conformations, the assessors consider five to be partial successes. However, for only one of these were high accuracy structures obtained for previously unknown conformational states, and all had partial templates. The one reasonably high accuracy result is for a ligand‐bound state of a porin, target T1214, where strands of the beta barrel structure bend into the barrel to make contacts with the ligand. There are low sequence identity homologs for this state present in the PDB, though. Overall, the results confirm that the state of the art in this area is still very poor. A variety of methods were used by participants, often based on some version of AlphaFold, but also sometimes outperforming AlphaFold3.

For the first time, CASP16 included a continuous conformational ensemble challenge for two sequences of the six‐residue flexible linker between two domains of Staph Aureus Protein A. It is not clear whether the features of this ensemble have functional significance, but a residual dipolar coupling (RDC) study [44] has shown that the distribution for the native linker is far from uniform. So, the system provides a good test for methods that attempt to predict such distributions. RDC and SAXS data were obtained for the two linker sequences considered, and assessment [45] was made in terms of the agreement of the calculated experimental data and those observed, and to the kernelized continuous representation [44] derived from the RDC data. Participants were invited to submit up to 1000 conformations of the system, together with occupancies for each. A wide variety of methods were used. Some groups produced a small number of conformations using AlphaFold3, and others submitted the maximum permitted 1000 conformations, often produced by approximate molecular dynamics‐based methods, some tuned to fitting SAXS data. None of the 35 submissions came close to matching both types of experimental data. But the study has identified a number of interesting questions. For example, the predictions with the closest fit to the SAXS data are not highly ranked when compared with RDC data. Also unclear is the impact of experimental uncertainty, both stochastic and systemic (e.g., the effects of the SAXS water model). Still, this is a major pioneering study, and it sets the stage for further development of assessment approaches.

Also for the first time, CASP16 included a challenge to reproduce the properties of a highly disordered system, the solvent density (water and ions) around a ribozyme structure (target R1260) [46]. Twelve groups made a total of 18 submissions, each typically consisting of an ensemble of more than 100 coordinate sets. Submissions were converted to an average electron density across each ensemble, and the density was compared to that obtained in two independent cryo‐EM studies, using four different metrics. Comparison of the results from the two experimental studies provided an estimate of the maximum achievable agreement. Computed solvent density agreement with experiment is considerably below that maximum, but there are multiple factors that may affect that besides the quality of the simulation: exact solvent composition, how density is generated from coordinate sets, the effect of RNA motion, the effect of radiation damage, among others. The assessors considered all these factors thoroughly, but the study highlights the difficulties of this type of assessment.

## 3 Conclusions

In the previous two CASPs, we saw enormous progress in computing macromolecular structures and complexes. First, in the 2020 CASP14, the arrival of AlphaFold2 resulted in the achievement of accuracy rivaling experiment for many protein monomers. Then, in the 2022 CASP15, the effective application of deep learning to protein complexes produced a huge leap in accuracy, though probably not quite rivaling experiment. We also saw some promising results for macromolecular conformational ensembles. In contrast to that, things seem to have stalled in CASP16. There is little sign of a closer approach to experiment for protein complexes. Both new and old RNA structure methods are woefully inadequate when there are no templates available, and results from macromolecular ensembles are generally disappointing. In the new category of protein–ligand interactions, results show a substantial improvement over a traditional method run as a control but will not put the experimentalists out of business. However, the medicinal chemistry refinement target datasets are substantially easier for deep learning methods than for docking newly discovered molecules to new targets [32].

So, is the deep learning revolution in computational structural biology over? There are strong grounds for expecting we will see further progress soon, some by the next CASP, from three types of advance.

The first is through the acquisition of more training data for the deep learning methods. No category currently has as much data to train on as protein monomers, so it is reasonable to expect that at least some categories are data limited. Prospects for more data vary. For protein–ligand complexes, OpenBind (https://www.gov.uk/government/news/uk‐to‐become‐world‐leader‐in‐drug‐discovery‐as‐technology‐secretary‐heads‐for‐london‐tech‐week) has the ambitious goal of obtaining 500,000 experimental complexes in the next 5 years. Target 2035 project (https://www.thesgc.org/target2035) also expects to generate a large number of complexes, although fewer will have structure. OpenFold3 [47] is working with pharma companies to gain access to their private data for method training, though the data will not become public. Direct experimental data in other categories may not grow nearly as quickly—the cost of new RNA structures is still substantial and the rate of production is low (in 2024 the PDB reports release 116 RNA‐only targets). But two other approaches to data extension hold some promise. The first is by self‐training [48]. That is, running existing methods on many samples (many RNAs expected to have structure for instance), selecting those estimated to be of high accuracy, adding those to the training data, retraining, and repeating the procedure. Although there are obvious risks, this approach has been effective in other areas. Second, in some cases it may be possible to add synthetic data to the training. It has been suggested that this approach may work for protein–ligand complexes using molecular dynamics to predict affinities, for example [49].

The second type of advance is through the combination of traditional physics‐inspired methods with deep learning. As noted earlier, in this CASP we saw encouraging signs that this will yield benefits. The most successful method for computing immune complexes [10] included a traditional docking method to augment an extensive sampling regime. Apparently, that way of generating possible starting structures sometimes helps overcome the inherent difficulty of this class of target. For RNA, the most successful group [11] combined a physics‐based method with AlphaFold3. These initial attempts at hybrid methods are likely to be developed further.

The third type of advance is improved algorithms. We have already seen the application of a whole range of deep learning techniques, starting with convolutional neural networks, then transformer/attention methods, large language models, and diffusion methods. We have also seen the introduction of many forms of structure representation, including graphs, grids, and interaction types. New combinations of these as well as new approaches will surely emerge. We will have to wait for the next CASP to see which of the likely developments pay off.

Most of the methods used in this CASP were at least partially built around AlphaFold ones, although some groups substantially modified those architectures. In most categories, some groups managed to do at least a little better than AlphaFold, primarily through the use of more extensive and strategic sampling, more careful engineering of multiple sequence alignments, clustering, and, as outlined above, combining with physics‐related methods. So far, though, none have really left the AlphaFolds in the dust.

## Author Contributions

**Andriy Kryshtafovych:** conceptualization, methodology, software, data curation, formal analysis, investigation, validation, visualization, resources, writing – review and editing, project administration. **Torsten Schwede:** conceptualization, methodology, software, data curation, formal analysis, writing – review and editing, investigation. **Maya Topf:** conceptualization, methodology, software, formal analysis, writing – review and editing, investigation. **Krzysztof Fidelis:** conceptualization, writing – review and editing, funding acquisition, investigation, resources, project administration. **John Moult:** conceptualization, methodology, data curation, investigation, validation, formal analysis, funding acquisition, visualization, project administration, writing – original draft.

## Conflicts of Interest

The authors declare no conflicts of interest.

## Supporting information


**Figure S1:** Percent of protein monomer evaluation units with backbone root mean square difference between computed and experimental backbones below 3, 2, and Å for the most recent three CASPs. By this metric, there is no increase in computed structures closer than 1 Å, consistent with agreement converging to the experimental limit. (Data are for the lowest RMSDs achieved on each target).
**Figure S2:** Average main chain agreement between closest computed and experimental protein evaluation units (using the GDT_TS metric) for non‐membrane spanning (non‐TM, blue) and membrane spanning targets in recent CASPs. After CASP12, there is no significant difference between the two classes.
**Figure S3:** Average main chain agreement between closest computed and experimental protein evaluation units (using the GDT_TS metric) for non‐viral and viral targets. There appears to be a small difference in accuracy for these two classes after CASP13.
**Figure S4:** Spread of Massivefold results for monomeric protein targets, sorted by LDDT range. Accuracy estimation evaluation (Section 2.5) was done on the target subset with a range of at least 0.2 LDDT units (boxed). IQR, inter‐quantile range.
**Figure S5:** An example of an ambiguous conformational ensemble result for the HIV‐1 Rev. Response Element Stem‐Loop II (SLII) conformational switch (R1203). There were two experimentally observed conformations (green and cyan). The closest submitted structure (magenta) lies between the two experimentally observed ones. Is this an incorrect model, or is there a continuum of RNA conformations that includes it?

## Data Availability

Data used in this work is freely available on the CASP website.
