# Supplementary material for: Progress and Bottlenecks for Deep Learning in Computational Structure Biology: CASP Round XVI
Source: Proteins. 2025 Nov 3;94(1):5–14. doi: 10.1002/prot.70076 (PMC12703882; doi:10.1002/prot.70076)
Supplement: Supplementary file 1 — Figure S1: Percent of protein monomer evaluation units with backbone root mean square difference between computed and experimental backbones below 3, 2, and Å for the most recent three CASPs. By this metric, there is no increase in computed structures closer than 1 Å, consistent with agreement converging to the experimental limit. (Data are for the lowest RMSDs achieved on each target). Figure S2: Average main chain agreement between closest computed and experimental protein evaluation units (using the GDT_TS metric) for non‐membrane spanning (non‐TM, blue) and membrane spanning targets in recent CASPs. After CASP12, there is no significant difference between the two classes. Figure S3: Average main chain agreement between closest computed and experimental protein evaluation units (using the GDT_TS metric) for non‐viral and viral targets. There appears to be a small difference in accuracy for these two classes after CASP13. Figure S4: Spread of Massivefold results for monomeric protein targets, sorted by LDDT range. Accuracy estimation evaluation (Section 2.5) was done on the target subset with a range of at least 0.2 LDDT units (boxed). IQR, inter‐quantile range. Figure S5: An example of an ambiguous conformational ensemble result for the HIV‐1 Rev. Response Element Stem‐Loop II (SLII) conformational switch (R1203). There were two experimentally observed conformations (green and cyan). The closest submitted structure (magenta) lies between the two experimentally observed ones. Is this an incorrect model, or is there a continuum of RNA conformations that includes it? [file PROT-94-5-s001.docx]

**SUPPLEMENTARY MATERIAL**

Supplementary Figure 1: Percent of protein monomer evaluation units with backbone Root Mean Square Difference between computed and experimental backbones below 3, 2, and 1 Angstroms for the most recent three CASPs. By this metric, there is no increase in computed structures closer than 1 Angstrom, consistent with agreement converging to the experimental limit. (Data are for the lowest RMSDs achieved on each target).

Supplementary Figure 2: Average main chain agreement between closest computed and experimental protein evaluation units (using the GDT_TS metric) for non-membrane spanning (non-TM, blue) and membrane spanning targets in recent CASPs. After CASP12, there is no significant difference between the two classes.

Supplementary Figure 3: Average main chain agreement between closest computed and experimental protein evaluation units (using the GDT_TS metric) for non-viral and viral targets. There appears to be a small difference in accuracy for these two classes after CASP13.

Supplementary Figure 4: Spread of Massivefold results for monomeric protein targets, sorted by LDDT range. Accuracy estimation evaluation (section 2.5) was done on the target subset with a range of at least 0.2 LDDT units (boxed). IQR is inter-quantile range.

### Supplementary Figure 5: An example of an ambiguous conformational ensemble result for the HIV-1 Rev Response Element Stem-Loop II (SLII) conformational switch (R1203). There were two experimentally observed conformations (green and cyan). The closest submitted structure (magenta) lies between the two experimentally observed ones. Is this an incorrect model, or is there a continuum of RNA conformations that includes it?
